# Validating Natural Compounds as Toll-Like Receptor 4 (TLR4) Antagonists: Experimental Challenges and Therapeutic Perspectives

**DOI:** 10.1021/acs.jmedchem.5c02252

**Published:** 2025-12-19

**Authors:** Federico Lami, Alessio Romerio, Laura Valle-Gómez, Olmo Martín-Cámara, Sonsoles Martín-Santamaría, Francesco Peri

**Affiliations:** † Department of Biotechnology and Biosciences, University of Milano-Bicocca, Piazza della Scienza, 2, Milano 20126, Italy; ‡ Centro de Investigaciones Biológicas Margarita Salas, CSIC, C/Ramiro de Maeztu 9, Madrid 28040, Spain

## Abstract

The activation of TLR4 by endogenous damage or danger-associated molecular patterns (DAMPs) suggested the use of TLR4 antagonists to target acute and chronic inflammatory diseases. In recent years, hundreds of natural compounds (NCs) have been screened for their activity as TLR4 antagonists. However, the direct interaction with TLR4 or TLR4/MD-2 dimer has not been proven for most of the natural molecules, and their mechanism of action has been only partially investigated. We review the recent literature on NCs active as TLR4 antagonists, analyzing the limitations and selecting among all candidates the compounds presenting new scaffolds desirable for drug development. After selecting a collection of representative hit structures, we also present novel docking calculations on TLR4/MD-2. Our analysis allowed the detection of common binding motifs in the receptor and the proposal of a structure–activity relationship from the ligands, enabling the discovery of new ligand scaffolds and pharmacophores for further development of drug hits.

## Graphical abstract

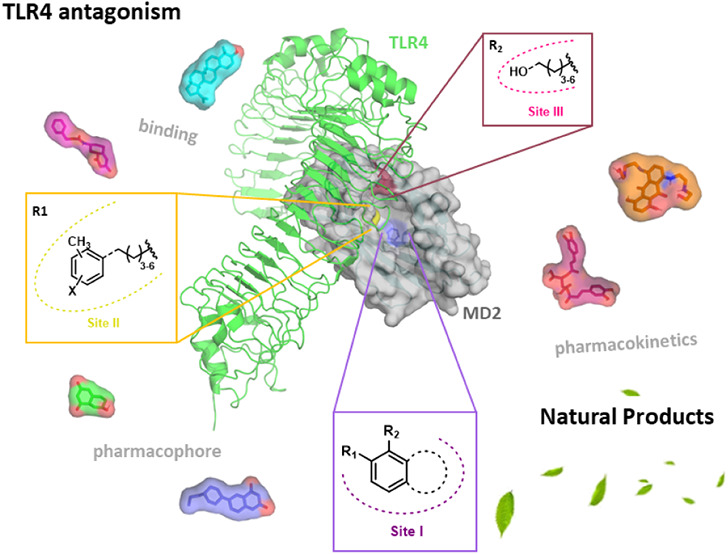

## Significance

- This Perspective paper presents the state-of-the-art knowledge about the use of NCs to modulate human TLR4 activation and signaling.
- It stimulates in readers a critical analysis of the literature data showing the properties of NCs as TLR4 ligands, helping them to understand the nature of TLR4 modulation and the rationale that underlies the selection of molecules targeting TLR4.
- It suggests new strategies to discover drug hits targeting the TLR4 activation.

## The Role of TLR4 in Diseases and Pharmacological Relevance of TLR4 Antagonists Discovery

Toll-like receptors (TLRs) have a primordial role in the activation of the innate immunity through the recognition of pathogen-associated and damage-associated molecular patterns (PAMPs and DAMPs), and they have sparked great interest in the therapeutic modulation of the innate immune system. Among them, Toll-like receptor 4 (TLR4) is one of the key receptors of innate immunity in mammals and humans, and it is the most extensively characterized in terms of signaling pathways and modulators. Its ability to recognize minute amounts of circulating endotoxin from Gram-negative bacteria (i.e., lipopolysaccharide, LPS or lipooligosaccharide, LOS, both in the form of aggregates or vesicles) makes this receptor one of the main sensors of bacterial infection.^1–4^

The recognition process of LPS by TLR4 situated in the plasma membrane and the subsequent intracellular signal activation have been widely investigated from both the functional/biochemical and structural/molecular perspectives.^5^ TLR4 extracellular domain (ectodomain) binds LPS assisted by two LPS-transfer proteins, LBP and CD14,^5–7^ and with the essential contribution of the MD-2 protein, which hosts the glycolipid portion of the LPS (lipid A, Figure 1A). The formation of the (TLR4/MD-2/LPS)_2_ heterodimer brings into proximity the intracellular Toll/interleukin-1 receptor (TIR) domains. Adaptor proteins subsequently bind to these domains, inducing the intracellular cascade, leading to the production of cytokines and inflammatory mediators. TLR4 is the only TLR receptor that, once activated by its ligands, can activate two different signaling cascades (Figure 1B). The first one, the MyD88-dependent pathway, engages the adaptor proteins TIRAP and MyD88, leading to the activation of the nuclear effector NF-κB and the consequent synthesis and release of various proinflammatory cytokines, among which are IL-1α, IL-1β, IL-6, and TNF.^2,8^ The second involves the adaptor proteins TRAM and TRIF and begins in early endosomes after LPS-induced endocytosis of the TLR4 mediated by CD14.^9^ The internalization of the CD14/TLR4/MD-2/endotoxin complex induces TRIF-dependent signaling, with subsequent interferon-β production.^2,10^

**Figure 1:**
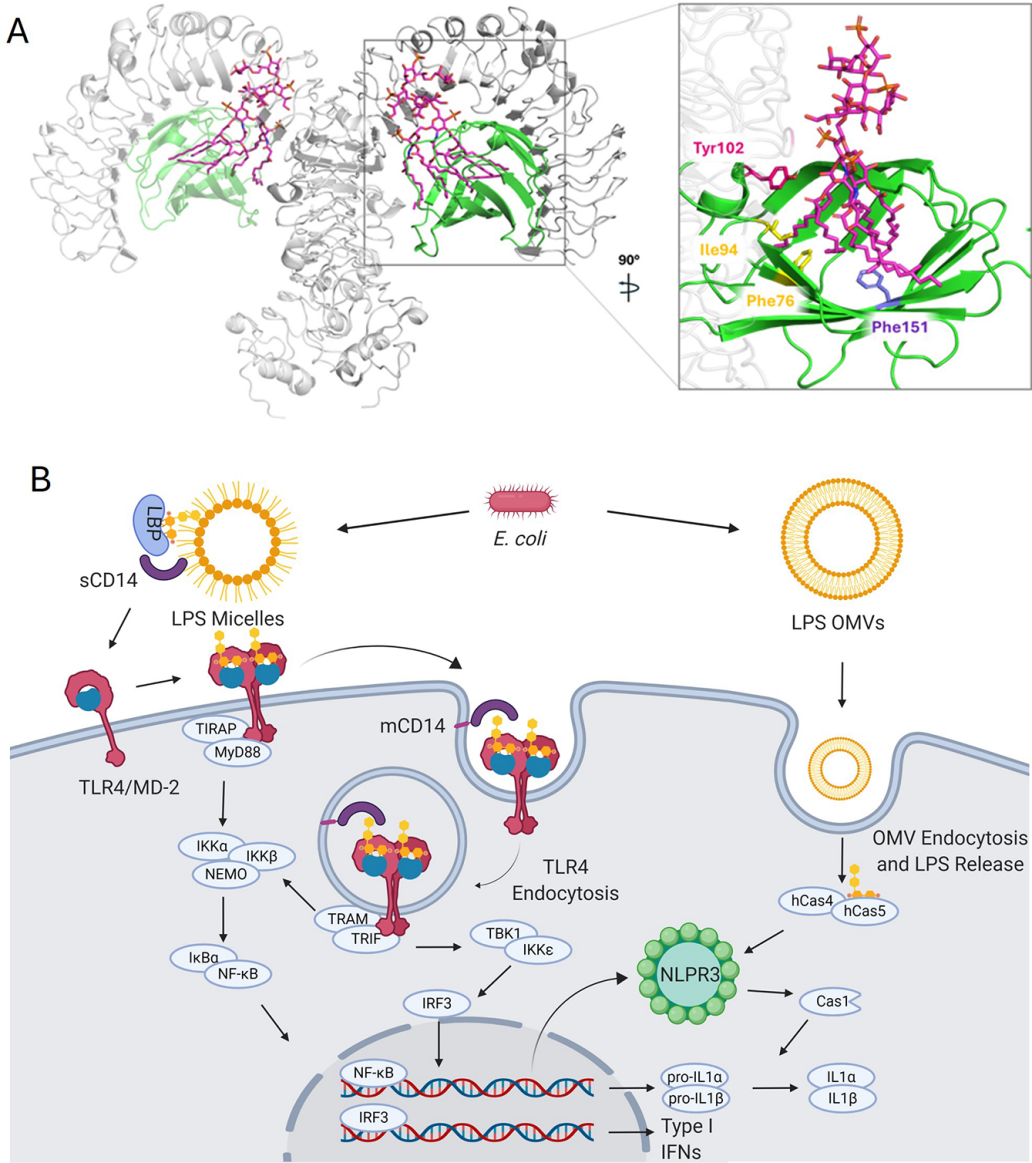
A) 3D structure of the (TLR4/MD-2)_2_ heterotetramer in complex with *Escherichia coli* LPS from PDB 3FXI (left). Details of the interaction between the lipid chains of lipid A from LPS and MD-2 (right). TLR4 is shown in gray. MD-2 is shown in green. LPS is shown in magenta sticks. B) Proinflammatory intracellular signaling pathways of TLR4 (MyD88 and TRIF/IRF3) and the NLRP3 inflammasome pathway initiated by LPS. From: Romerio, A.; Peri, F. Increasing the Chemical Variety of Modulators: An Overview. *Front Immunol* **2020**, *11* (July), 1–16.

From a pharmacological point of view, TLR4 has been considered the main target for Gram-negative bacterial sepsis caused by circulating LPS. Indeed, a dysregulated and excessively potent TLR4 activation by endotoxin is the underlying cause of the multiple organ failure in septic shock.^11^ Sepsis is one of the most common causes of death in the world: in 2017 alone, there were 11 million reported cases of sepsis-related death. Notwithstanding, there is no specific pharmacological treatment yet.^12,13^ Also some PAMPs from other infectious agents, as viruses, are recognized by TLR4 and can cause an excessive immune response very similar to acute sepsis, leading to lethal cytokine storm.^14^ An example is the family of filoviruses (e.g., Ebola, Marburg), whose secreted glycoproteins act as TLR4 ligands, activating the inflammatory cascade.^15,16^ It has been recently shown that administering TLR4 antagonists to mice challenged with Ebola can reduce the pathogenesis and lethality of the virus.^17,18^

Additionally, the perspective to target TLR4 antagonists sparked great interest due to the observation that TLR4 can also be activated by endogenous DAMPs, for example, proteins such as HMGB1 and IFI16 ^19–21^ or aberrant metabolites such as oxidized LDL and oxidized phospholipids.^22–24^ Indeed, the DAMP/TLR4 axis has been linked to a series of acute and chronic inflammatory syndromes called sterile inflammations because of the lack of an infectious agent as a trigger. Chronic inflammation in inflammatory bowel diseases (Crohn’s disease, ulcerative colitis),^25^ atherosclerosis,^26–28^ diabetes,^29^ ocular diseases,^30^ autoimmune syndromes such as rheumatoid arthritis^31^ and lupus^32^ are generated by TLR4 (over)­stimulation by DAMPs.^3,28–38^ In the same way, excessive TLR4 activation by DAMPs is also key to neuroinflammation and neurodegenerative diseases such as Alzheimer’s disease (AD),^29,39,40^ amyotrophic lateral sclerosis (ALS),^39,40^ or Parkinson’s disease (PD).^38,41,42^

Notwithstanding the pivotal role of TLR4 in inflammation, only two compounds have advanced to a phase III clinical trial: Eritoran (developed by Eisai, Figure 2) and TAK-242 (developed by Takeda, Figure 2). Even though both compounds failed in clinical phase III as antisepsis agents, they have been tested in clinical trials for other inflammatory pathologies.^43,44^ Eritoran has been tested in phase II against lipid-dependent insulin resistance without reaching the desired end points.^45^ It is currently in phase III against community-acquired pneumonia (REMAP-CAP, to be completed in 2028).^46^ TAK-242 is currently in two phase II clinical trials against severe alcoholic hepatitis and acute-on-chronic liver failure, either alone or in combination with G-CSF (A-TANGO, to be completed in 2026).^47,48^

**Figure 2:**
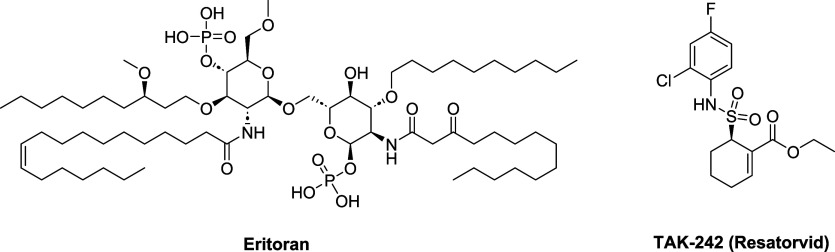
TLR4 antagonists in clinical trials.

Natural and synthetic molecules are in preclinical development for PAMPs- and DAMPs-related pathologies. For example, synthetic FP compounds were effective in blocking lethality linked to acute respiratory distress syndrome (ARDS) and acute lung injury (ALI) in viral airway infections.^49–55^ Natural small molecule 6-shogaol treatment prevented articular cartilage lesions, synovitis, and the presence of proinflammatory mediators and disease markers in osteoarthritis animals by TLR4 inhibition.^56^ Ferulic acid, another natural small molecule, proved to be able to protect mice from LPS-induced acute kidney injuries.^57^

Studies of the 3D molecular recognition at the atomic level of the TLR4/MD-2/ligand complex are of paramount importance for understanding the mechanism of action and for designing new modulators with improved affinity. Due to the high complexity of the TLR4/MD-2 system and its peculiar features as a noncanonical TLR, the approach to such studies through experimental techniques, such as X-ray crystallography, NMR, or cryo-electron microscopy (cryo-EM), has yielded successful examples with only few X-ray crystal structures of TLR4/MD-2/ligand complexes that inform us about the most important features of the binding mode (Figure 1A).^58,59^ Moreover, computational techniques have provided interesting and useful insights to rationalize the agonist/antagonist mechanism and to advance the design of TLR4 modulators by combining docking and molecular dynamics simulations.^59,60^

## Natural Compounds (NCs) Targeting TLR4: Going Beyond the State of the Art

NCs are a source of chemical diversity in drug discovery, and several NCs have been investigated for their capacity to inhibit TLR4 activation. Several natural and synthetic compounds can downregulate the complex process of TLR4 activation with different mechanisms of action and target different points in the pathway. For example, they can inhibit the TLR4/MD-2 dimerization by binding MD-2 or TLR4 at the dimer interface, preventing ligand binding or disrupting the TLR4/MD-2 dimer.^61,62^ Other compounds can instead target another region on TLR4 or an intracellular protein activated downstream to TLR4 or even downregulate TLR4 expression in the first place.^63,64^

Despite these advances, the integration of experimental and computational data remains limited for many NCs. The heterogeneity in assay conditions and modeling protocols complicates direct comparison among studies, underscoring the need for standardized protocols and comprehensive validation. The identification of natural TLR4 antagonists with well-defined binding modes and high binding affinity continues to be a critical bottleneck in translating natural product scaffolds into therapeutic candidates. More in detail, the literature data available suffer from the following limitations:^65^

Very often, published works report on indirect evidence of TLR4 targeting, based on biological readouts (e.g., NO and cytokine production, reduction of inflammation in cell or animal models after LPS challenge) not directly related to TLR4 antagonism. No direct binding experiments among the natural molecules and the receptor dimer TLR4/MD-2 through techniques such as surface plasmon resonance (SPR), isothermal calorimetry (ITC), fluorescence or nuclear magnetic resonance (NMR) experiments, or TLR4 reporter cell assessments are reported. The properties observed may therefore be caused by the inhibition of other downstream proteins, such as NEMO or TBK1, or by off-target effects such as the inhibition of other pattern recognition receptors (PRRs).
The effect of an extract of natural origin (plant, fruit, etc.), which is a complex mixture of molecules, is often tested without subsequent characterization of the TLR4 activity of the single components; therefore, it is impossible to pinpoint the molecule(s) responsible for the activity. For the same reason, it is not possible to exclude off-target effects on other PRR on or downstream effectors, as in the previous case.
Rationalization of the TLR4/MD-2-mediated mechanism by computational techniques for further structure–activity relationship (SAR) conclusions and computer-aided drug design is currently hindered by the heterogeneity of existing computational studies. While some investigations employ state-of-the-art computational procedures by combining docking/simulation approaches with in-depth analysis of ligand–receptor interactions and binding modes, others suffer from nonrigorous computational protocols, including the use of nonrefined X-ray crystal structures, the selection of incorrect human or animal sequences, and wrong assignment of agonist or antagonist conformations of the TLR4/MD-2 complex.^66–68^ Such inconsistencies compromise the reproducibility and reliability of SAR insights and highlight the urgent need for standardized, high-quality computational frameworks in this field.

Several NCs recently emerged as TLR4 antagonists: anthraquinones such as Emodin, Aloe Emodin, Rhein, 2-hydroxy anthraquinone, 2-carbomethoxy-2,3-epoxy-3-prenyl-1,4)­naphthoquinone, and their semisynthetic derivatives Mitoxantrone, Pixantrone, and Mitoxantrone­(2-hydroxyethyl)­piperazine (Mitoxantrone metabolite);^69–74^ astragaloside IV saponin,^75–95^ Lupeol,^96–104^ and Ginsenosides extracted from Ginseng (G-Rb1, G-Rb2, G-Re, C-k, and G-Rt_8_);^105–119^ cichoric acid;^120–125^ dihydroartemisinin;^125–134^ Dysodensiol K;^137^ and Mangiferin.^136–145^ However, many studies suffer from the limitations listed above, and the anti-inflammatory properties of the tested compounds may not be related exclusively to TLR4 antagonism.

This perspective presents a subgroup of these antagonists, whose chemical structures are represented in Figure 3, according to the following selection criteria: 1) antagonists in the more stringent definition, so molecules that have shown to inhibit LPS-dependent TLR4/MD-2 dimer formation and subsequent intracellular signaling; 2) original scaffolds, excluding classes of compounds known since a long time and exhaustively reviewed by us and others;^3,35,42,58,59,146–149^ 3) compounds that have in general low toxicity tested in animals and present good pharmacodynamic properties; 4) compounds with a 3D perspective of their TLR4/MD-2 binding by revising and revisiting computational molecular docking calculations. The combination of these properties makes these structures interesting drug hits for further preclinical development.

**Figure 3:**
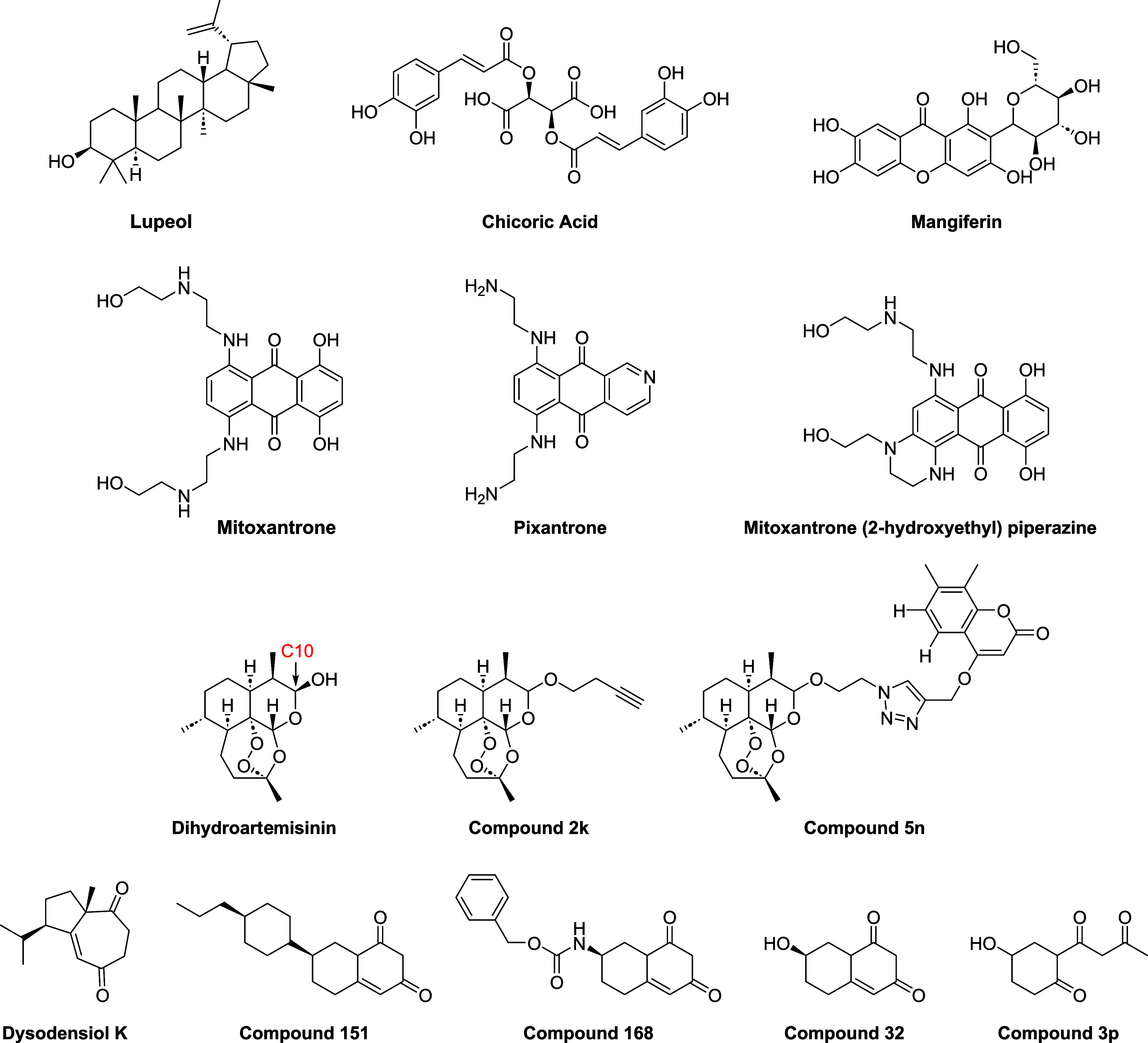
Chemical structures of the NC active as TLR4 antagonists that are described in this Perspective.

## Recently Reported NCs with TLR4 Antagonist Activity: Looking for New Scaffolds and Pharmacophores

**Lupeol** (Figure 3) is a pentacyclic triterpene found in extracts of medicinal plants such as licorice or *Emblica Officinalis*, as well as in common vegetables (white cabbage, pepper) or fruits (mango, strawberries).^99^ In the past, its anti-inflammatory, antiapoptotic, antioxidant, and anticancer properties have been highlighted, but despite its lack of toxicity (nontoxic *in vivo* up to a 2000 mg/kg dose), Lupeol’s pharmacodynamic properties are yet largely unknown.^96,97,101^ The reported action of Lupeol in mice models of neuroinflammation and the calculated log*P* 8.4 suggest good distribution and pharmacokinetic properties, as well as the capability to penetrate the blood–brain barrier (BBB) and enter the central nervous system (CNS). Lupeol showed neuroprotective effects both *in vivo* and *in vitro*. Administration of 50 mg/kg/day alleviated the LPS-induced neuroinflammation in mice and reduced the activation of TLR4-dependent p38 and JNK pathways, inhibiting the production of proinflammatory markers such as TNF, NOS-2, and IL-1β. It attenuated microglia and astrocytes activation and reduced apoptosis.^103^ Lupeol showed to be effective in alleviating traumatic brain injury (TBI) symptoms *in vivo* as it reversed glial cell activation and reduced oxidative stress, neuroinflammation, apoptosis, and memory impairment in mice.^104^ Lupeol reduced the mRNA expression of iNOS, TNF, and NLRP3 inflammasome, as well as NO production, *in vitro* in cerebellar-derived astrocytes, at a concentration of 0.1 μM.^104^ The cardioprotective effects of this triterpene were evidenced in recent years since it proved to be effective in alleviating coxsackie virus B3 (CVB3)-induced myocarditis *in vivo* subsequently to an administration of 50 mg/kg, and the involvement of the TLR4/MyD88/NF-κB p65 pathways was determined as the expression of these proteins was reduced upon treatment.^100^ Lupeol showed heart-protective effects since it was able to reduce IL-6, IL-10, and TNF levels via the TLR4/PI3K/Akt/NF-κB pathway both *in vitro*, in neonatal rat cardiomyocytes (NRCMs) at a concentration of 50 μg/mL, and *in vivo* following a treatment of 50 mg/kg/day.^98^ Furthermore, Lupeol was able to reduce retinal inflammation in a model of LPS-induced uveitis both *in vivo* and to reduce proinflammatory cytokines (IL-8 and IL-6) levels *in vitro* in human retinal pigment cell line (ARPE-19) at a concentration of 100 μM.^96^

### Anthracycline Derivatives

Mitoxantrone (Figure 3), a semisynthetic anthracycline derivative, is a polypharmacological immunosuppressive drug used in the treatment of different cancer types since 1978.^71,72^ The main issue with this drug is its cardiotoxicity, and therefore new less toxic derivatives have been developed, such as Pixantrone and Mitoxantrone (2-hydroxyethyl)­piperazine (a Mitoxantrone metabolite, Figure 3).^71^ Recently, the immunosuppressive mechanism of this class of molecules has been investigated: the results showed that Mitoxantrone, Pixantrone, and Mitoxantrone­(2-hydroxyethyl)­piperazine act as TLR4 antagonists by inhibiting NF-κB translocation in HEK-Blue-hTLR4 cells and decreasing TNF production in neonatal mouse microglia cells.^71^ Even though the anthracycline derivatives are too toxic to be considered as drug leads, their strong TLR4 antagonistic activity suggests a high affinity for the receptor. Given that there are no reported computational or structural studies of the TLR4 binding for these drugs, and the evidence of direct TLR4 binding according to the HEK-Blue-hTLR4 cells studies, we decided to include Mitoxantrone in our docking calculations as a reference TLR4 antagonist (see below).

### Cichoric Acid (CA)

Dicaffeoyl-tartaric acid, also known as cichoric acid (Figure 3), is a naturally occurring polyphenol belonging to the hydroxycinnamic acids class, present in the extract of various medicinal plants, such as chicory (*Cichorium intybus*), dandelion (*Taraxacum officinale*), and echinacea (*Echinacea purpurea*).^121^ The anti-inflammatory and antioxidative effects of CA are known, but only in recent years, they have been investigated in more detail. For example, its liver-protective effect has been proven *in vitro* and *in vivo*, since it was able to reduce MyD88, iNOS, and TNF expression in LPS-induced RAW264.7 cells at a concentration of 64 μM and to protect mice against ethanol-induced liver steatosis upon treatment with 4 mg/kg/day.^121^ Furthermore, CA regulated autophagy in HepG2 cells and inhibited MyD88 and NLRP3 expression at a concentration of 50 μM, to protect mice from LPS/d-galactosamine-induced acute liver failure with a treatment of 12.5, 25, and 50 mg/kg/day.^122^ CA proved to be effective in protecting BV2 microglia cells from LPS-induced inflammation, reducing NO and ROS production at a concentration of 80 μM.^120^ CA antagonistic activity of TLR4 has been shown *in vivo* in another study, in which it inhibited IL-6 and IL-1β levels in mice serum, striatum, and colon both at 30 mg/kg/day and 60 mg/kg/day via downregulation of the TLR4-dependent pathway, protecting brain and gut from Parkinson disease symptoms.^123^ The anti-inflammatory/antioxidant activity of CA proved to be useful in the reduction of LPS-induced inflammation in yak peripheral blood mononuclear cells (PBMCs), as it lowered the expression of IL-6, IL-8, IL-1β, IFN-γ, and TNF by targeting the TLR4/MyD88/NF-κB signaling at 60 μg/mL.^150^ CA alleviated inflammation in bovine lamellar keratinocytes at a concentration of 120 μg/mL, as it reduced proinflammatory cytokines (IL-6, IL-1β, and TNF) expression as well as TLR4 and MyD88 expression.^125^ CA ameliorated high-purine diet-induced hyperuricemia in quails by regulating gut microbiota and reducing proinflammatory cytokines expression: treatment with 16.78 mg/kg/day proved to be effective in lowering IL-6 and TNF production via inhibition of TLR4/MyD88/NF-κB pathway.^151^ Recently, CA has been effectively incorporated in folate-functionalized liposomes and tested in an ulcerative colitis model.^152^ This formulation was tested *in vitro* in RAW264.7 cells at a concentration of 100 μg/mL, where it was active in downregulating iNOS and CD86 levels as well as mRNA expression of proinflammatory cytokines (IL-6, IL-1β, TNF), in upregulating anti-inflammatory factors such as IL-10 and CD86 and in decreasing the M1/M2 polarization ratio of macrophages.^150^ *In vivo* administration of 10 mg/day of CA/folate liposomes resulted in the same effects as shown *in vitro*, except for the upregulation of IL-10.^152^

### Dihydroartemisinin

DHA (Figure 3) is the active metabolite of artemisinin, the main component of the *Artemisia annua* extract, a well-established and widely used antimalarial drug. The activity of artemisinin and its derivatives as TLR4 antagonists has been recently investigated *in vitro* and *in vivo*, in several TLR4-dependent inflammatory diseases.^126^ Interestingly, DHA showed unusual selective targeting toward the TLR4/IRF3/IFN-β pathway. This was demonstrated in a study on RAW264.7 murine macrophages, in which DHA reduced LPS-induced inflammation by selectively targeting the TRIF/IRF3 signaling and leaving the MyD88 pathway unaffected.^131^ This can lead to targeted therapy in which partial inflammation reduction without complete abolishment is desirable, such as in autoimmune diseases (e.g., rheumatoid arthritis or other autoimmune rheumatic diseases, such as systemic lupus erythematosus, SLE).^153^

Indeed, DHA showed beneficial effects in SLE treatment by reducing LPS-induced inflammation in spleen cells derived from SLE-prone mice via TLR4/IRF3 pathway inhibition. DHA also reduced the expression of the molecular mediators of SLE inflammation, namely TLR4/IRF3/IFN-β, in a dose-dependent manner in a concentration range from 0.1 μM to 10 μM.^130^ Recently, also a formulation comprising DHA co-delivered with HGMB1 siRNA in a PEG liposome functionalized with a cell-penetrating peptide was tested *in vitro* as anti-SLE treatment. This new formulation blocked LPS-stimulated NF-κB translocation in HEK-Blue cells at 100 nM; furthermore, it reduced the expression of HMGB1, TLR4, IRAK4, MyD88, NF-κB, and proinflammatory cytokines (IL-6, IL-1β, IL-8, TNF) in murine macrophages, as well as inhibited the proliferation of glomerular mesangial cells.^133^ Moreover, DHA protected mice by LPS-induced acute kidney injury by downregulating the NF-κB signaling and reducing oxidative stress.^129^ Furthermore, dietary administration of DHA to piglets affected by intrauterine growth retardation was shown to have gastro-protective activity via TLR4/NOD/NF-κB signaling, posing evidence that this molecule might not be entirely TLR4 selective.^132^ DHA showed neuroprotective effects deriving from TLR4 targeting, as it alleviated morphine-induced neuroinflammation in BV2 murine microglial cells via reducing the expression of TLR4 and inflammation-related proteins (IL-6, IL-1β, TNF).^127^ DHA was tested as a treatment for colitis-associated colorectal cancer (CAC), and it was effective in reducing inflammation and preventing M1 macrophage migration, both *in vitro* (RAW264.7, THP-1, HCT116, and RKO cell lines) and *in vivo* in a CAC mouse model. DHA proved to be useful in the late stages of cancer as it inhibited tumoral cell growth.^128^ Finally, DHA reduced the muramidase-released protein inflammatory response following infection with *Streptococcus suis*, both *in vitro* in RAW264.7 macrophages and *in vivo* in mice.^134^ Given the good efficacy of DHA as a TLR4 antagonist, and the growing interest in both natural molecules and anti-inflammatory compounds, several derivatives of this natural metabolite have been synthesized, aiming at increasing activity and selectivity toward TLR4. In a recent work, several DHA-coumarin covalent dimers have been developed and tested as antineuroinflammatory agents. Compound **5n** (Figure 3), in which a coumarin moiety is linked to DHA through a triazole-containing linker, was the most effective, as it reduced NO, IL-6, and TNF levels with an IC_50_ of 0.22 μM.^152^ A structure–activity relationship (SAR) study was carried out using DHA derivatives in which C10 hydroxyl was protected as an acetal, and prodrug **2k** (Figure 3) was evidenced as the most active. This molecule was able to reduce the production of IL-6, IL-1β, and TNF *in vitro* in BV2 microglial cells with an IC_50_ of 50 μM, as well as to enhance morphine analgesia in mice.^127^ Docking studies showed that the orientation of **2k** in the MD-2 pocket is reversed with respect to DHA, and the alkyl group in position C10 is deeply inserted in the MD-2 hydrophobic pocket.^154^ These data, taken together, suggest that the TLR4 antagonistic activity of DHA and its derivatives is tightly related to the interaction with the MD-2 coreceptor and that optimization of the binding with the entire TLR4/MD-2 dimer by adding moieties capable of interacting with TLR4 residues could maximize the pharmacodynamic properties of DHA derivatives.

### Dysodensiol K Derivatives

Dysodensiols are a class of molecules extracted from the roots, leaves, and fruits of *Fissistigma oldhamii*, an herb commonly used in traditional medicine. These compounds share a common scaffold comprising a seven-membered ring condensed with a five-membered ring and ketonic or hydroxylic moieties on different regions. Dysodensiol K (DK) (Figure 3), extracted from the roots, showed antirheumatoid arthritis (RA) properties, as it decreased the proliferation of rat synovial cells (RSC) with an IC_50_ of 11.8 μM.^135^ DK was used as a reference compound to study similar scaffolds, which, in turn, were obtained through high-throughput screening to evaluate the anti-RA activity of DK synthetic derivatives. Through SAR analysis, it emerged that a bicyclic structure displaying a conjugated enone is the pharmacophore of Dysodensiol K. This knowledge was exploited to design and obtain new structures.

In a first screening round, compounds **151** and **168** (Figure 3) showed the best properties: *in vitro,* they inhibited RSCs proliferation with IC_50_ values 2.71 μM and 2.68 μM, respectively; *in vivo*, they inhibited IL-6 and TNF production in mice upon treatment with 50 mg/kg/day, and were less cytotoxic than methotrexate, a common drug for RA treatment used as positive control. A following *in vitro* study showed that **168** and **151** exert their activity through TLR4 antagonism with IC_50_ values respectively of 0.56 μM and 0.73 μM: these compounds also inhibited downstream TLR4 effector as TNF, IL-6, and IL-1β in RSCs.^155^ Furthermore, **168** was able to downregulate the expression of the pro-apoptotic protein Caspase 3, as well as upregulate TLR4, MyD88, and NF-κB expression.^155^ As the activity of **168** and **151** was not significantly better than methotrexate, other optimization rounds have been carried out, resulting in compounds **32** and **3p** (Figure 3).^156,157^ **32** resulted in an increased activity with respect to methotrexate as well as lower cytotoxicity *in vitro* and lower acute toxicity *in vivo*. This compound inhibited RSCs proliferation with an IC_50_ of 1.36 μM, and TLR4 with an IC_50_ of 0.41 μM and decreased LPS-induced production of IL-6, IL-1β, and TNF in mice upon treatment with 50 and 100 mg/kg/day. Compound **3p** showed a lower IC_50_ (0.73 μM) in inhibiting RSCs proliferation but a slightly higher TLR4 IC_50_ (0.43 μM), and it reduced the pro-inflammatory cytokines in LPS-induced RAW264.7 macrophages.

### Mangiferin

1,3,6,7-Tetrahydroxyxanthone-C2-β-glycoside, or Mangiferin (MF, Figure 3), belongs to the xanthones family and is found in extracts from various plants, such as *Mangifera indica*, *Anemarrhena asphodeloides*, and *Rhizoma anemarrhenae* and in *Swertia* or *Coffea* species. MF-containing herbs are well known in Chinese traditional medicine and, in recent years, this xanthone has been considered one of the best candidates to treat inflammation-related diseases; however, its mechanism of action is yet to be fully understood.^136^ Mangiferin was tested for colitis treatment in LPS- or peptidoglycan-stimulated peritoneal macrophages, and it reduced IL-6, IL-1β, TNF, i-NOS, and COX-2 expression as well as IRAK1, MAPKs, JKN, ERK, p38 phosphorylation, and NF-κB activation upon treatment in a dose-dependent manner at 5, 10, and 20 μM. The same results were obtained *in vivo* in mice, where MF improved dextran sulfate sodium (DSS) or 2,3,4-trinitrobenzenesulfonic acid (TNBS)-induced colitis symptoms, reducing weight loss, colon shortening, and myeloperoxidase activity upon treatment with 20 mg/kg/day.^139,156^ MF also showed neuroprotective effects, as treatment with 50 mg/kg/day reduces LPS-induced inflammation in the hippocampal region of mice brain, downregulating IL-6 expression and reducing cognitive impairment, as well as upregulation heme oxygenase-1 (HO-1) levels.^140^ This natural active principle may be helpful in the treatment of periodontitis, as it reduced TLR2, TLR4, and IL-6 expression derived from LPS-induced inflammation in immortalized human oral keratinocytes (OKF6/TERT2) showing dose dependency at 10, 20, and 40 μM. It also inhibited phosphorylation of NF-κB, p38, MAPK, and JNK, proving to be a promising drug for the treatment of this illness.^145^ Mangiferin reduced pro-inflammatory cytokines (IL-6, IL-1β, TNF) expression, NF-κB and NLRP3 inflammasome activation in mice with LPS-induced mastitis, as well as improved mastitis-derived symptoms and MPO activity upon treatment with 5, 10, and 20 mg/kg.^141^ The antifibrotic effects of MF were evidenced in a study where treatment with 40 mg/kg MF reduced histopathological lesions and improved bodyweight, survival rate, and pulmonary index in mice with bleomycin-induced pulmonary fibrosis, via TLR4/p65 pathway inhibition. *In vitro* studies on A549 cells, using 10 μg/mL of MF, demonstrated that the antifibrotic effects are also due to TGF-β/Smad2 or Smad3 pathway inactivation.^141^ Mangiferin exhibited liver protective properties, as it reduced the expression of TLR4 and TNF as well as AP1 and NF-κB activity in LPS-stimulated Kupffer cells at 100 μM. Furthermore, MF was able to improve LPS or D-GalN-induced acute liver injury-related symptoms, to downregulate TNF expression and upregulate HO-1 levels in mice with a treatment of 40, 100, and 150 mg/kg.^143^ The anti-inflammatory activity of MF was proved again as it reduced *Staphylococcus aureus*-induced inflammation, apoptosis, and modulated g autophagy in RAW264.7 macrophages, via reduction of proinflammatory IL-6, IL-10, TNF, Bax, and Caspase3 levels and increasing Bcl-2 production in a dose-dependent manner at 25, 50, and 100 μM.^137^ In a following work, LPS-induced ALI complications in mice were alleviated upon pretreatment with 100 mg/kg of MF. Furthermore, *in vitro* studies in murine J774A-1 cells showed that this effect is due to the inhibition of NLPR3 inflammasome activation, via TLR4/p65 pathway inactivation.^138^ In a recent study, MF was coadministered with cinnamic acid to treat rheumatoid arthritis (RA) in rats, and it decreased RA symptoms severity via TLR4/PI3K/AKT/NF-κB signaling inhibition, leading to a suppression of NLRP3 inflammasome activity, downregulation of IL-1β and Caspase3 release, and modulation of GSDMD-mediated pyroptosis. *In vitro* analysis on RAW264.7 and MH7A cells confirmed the data obtained *in vivo*. Furthermore, MF anti-inflammatory activity was proved in a subsequent work, in which it inhibited LPS-induced inflammation in RAW264.7 macrophages and reduced sepsis in CLP-stimulated mice. *In vitro* data demonstrated that Mangiferin downregulates the expression of proinflammatory cytokines (IL-6, TNF) via TLR4/MyD88/NF-κB signaling inhibition. Moreover, MF was able to restore sepsis-derived intestinal flora imbalance.^144^

## Integrated TLR4/MD-2 Binding Studies by Molecular Docking Calculations

For some of the TLR4 antagonists reviewed here, docking calculations have been reported to explain the biological activity (measured, for example, as a decrease of IL-6, TNF-α, iNOS, and NO production) and to establish structure–activity relationships. These compounds are lupeol, cichoric acid, DHA and DHA analogue **2k**, Dysodensiol K analogues **151**, **168**, **32**, and **3p**, and Mangiferin, and the docking calculations were used for providing information about binding sites into TLR4 and the corresponding binding modes. However, the employed computational methodologies were not uniform, making it difficult to compare them and derive common conclusions. To ensure consistency and comparability across all compounds, we repeated the docking calculations for all of these compounds using our standardized protocol. These compounds also provide chemical diversity and can serve as the basis for studying binding modes and facilitating comparative analysis.

The first point to determine is the target protein to be considered as a macromolecule for reliable and meaningful protein–ligand docking. It is well established that TLR4 is associated with MD-2 and that this association is required to activate and inhibit intracellular signaling, even though we cannot rule out that TLR4 alone could be targeted by ligands and its activity could be inhibited in this way. Thus, since our focus is to rationalize the binding mode and activity properties to design and develop selective TLR4/MD-2 antagonists, our reference is the competition with the natural agonist, i.e., the LPS, that targets the TLR2/MD-2 dimer. However, many reported docking calculations exclusively focused on the TLR4 as target protein. Therefore, a proper approach for rational design would be to focus on the MD-2 hydrophobic cavity, where most of the agonists bind, such as many lipid A variants and synthetic or natural phosphoglycolipids.

Another difficulty in approaching the reported calculations is that they differ in the docking program used, making difficult the direct comparison among the proposed binding modes and scores since each docking program employs different algorithms for posing and scoring. Therefore, we present here a set of new homogeneous docking calculations of the TLR4 modulators. To this end, we have applied a common docking protocol to selected lead TLR4 antagonists from different NC families, facilitating a direct comparison in terms of binding sites and binding modes.

Given that there are no experimental 3D structures available for the human TLR4/MD-2 dimer in the antagonist conformation, we used the computational model reported by us as the macromolecule for the docking.^157^ The small molecules were built and optimized under OPLS4 force field with Maestro.^158^ Fully flexible docking calculations were performed with the help of AutoDockTools and VINA,^159,160^ by setting the grid box dimensions to 50 × 48 × 52 points with grid spacing of 1 Å along the *X*, *Y*, and *Z* axes, with the center positioned equidistantly from the mass centers of residues Leu78, Phe121, and Ile94 of MD-2. Upon exploring the resulting docked poses, we identified three binding sites (named sites I, II, and III, Figure 4A) based on the resultant calculations. Most of the docked poses were placed inside MD-2 by establishing hydrophobic interactions within the MD-2 pocket and polar interactions at the MD-2 rim and with TLR4.^161^

**Figure 4:**
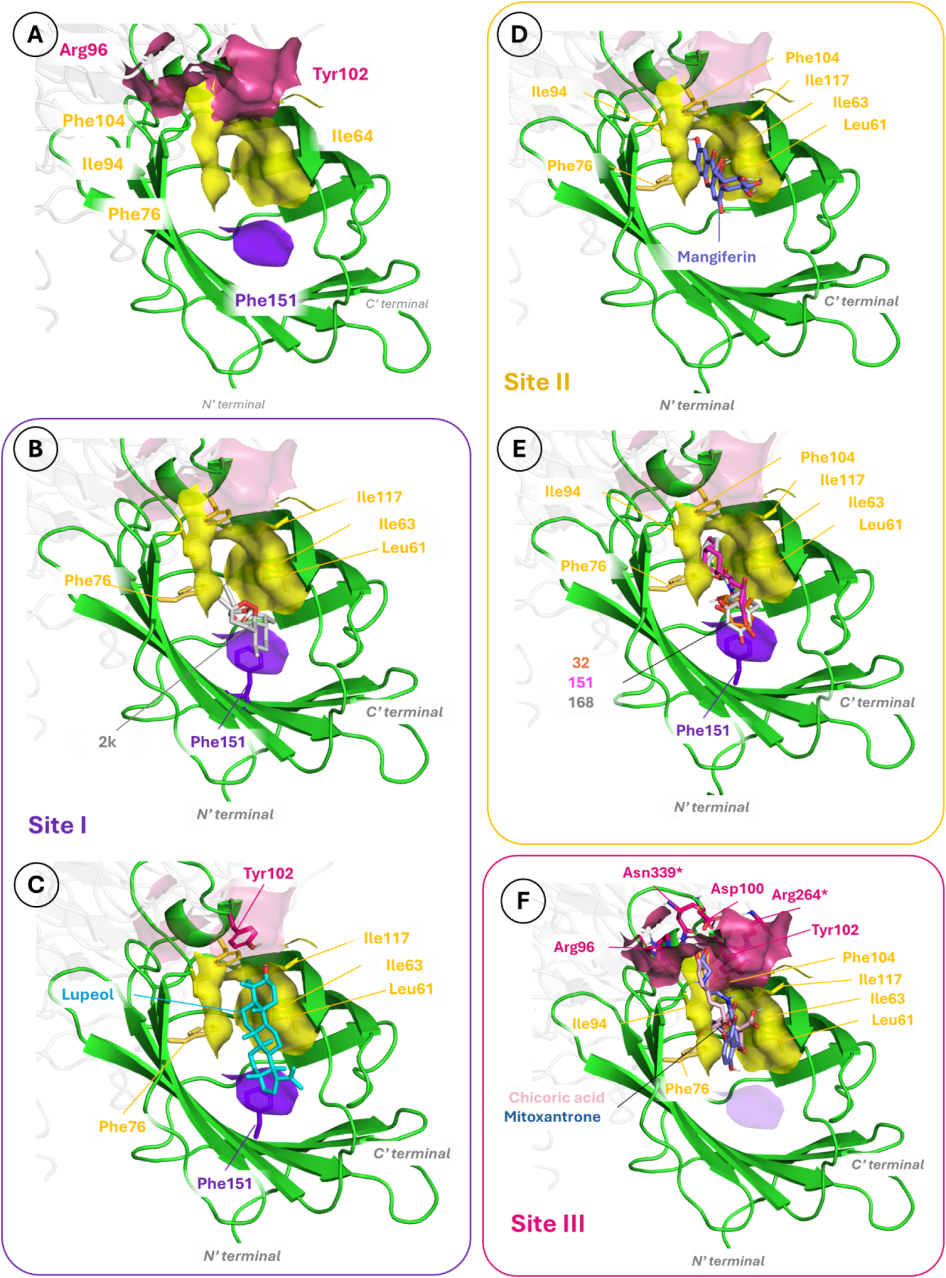
A) Binding sites identified as the preferred sites after docking calculations of the compounds inside MD2: site I (purple), site II (yellow), and site III (raspberry rose). Details of site I with the best docked solution for B) compound **2k** and C) **Lupeol**. Details of site II with the best docked solutions for D) **Mangiferin** and E) compounds **32** (orange), **151** (pink), and **168** (light gray) shown superimposed. Details of site III with the best docked solutions for F) **CA** (light pink) and **Mitoxantrone** (blue) shown superimposed. Nonpolar hydrogens have been omitted for clarity.

**Site I** is centered at Phe151, whose aromatic ring is involved in π–π interactions with aromatic rings of the ligands. Besides, van der Waals interactions between aliphatic surrounding amino acids (Leu61, Phe76, Ile94) as well as polar contacts with the polar rim of MD-2 (Tyr102, Glu92, Ser118, or Ser120) contribute to anchoring the ligands. The largest compounds, compound **2k** and Lupeol, preferentially bind to this site (Figure 4B,C), interacting with the above-mentioned nonpolar amino acids, even though they have a different calculated Log*P* (respectively, 3.42 and 7.27). Furthermore, Lupeol, being larger, occupies the MD-2 cavity more extensively and forms a hydrogen bond between its hydroxyl group and the Tyr102 hydroxyl group, further stabilizing this binding mode. **Site II** comprises Leu61, Ile63, Phe76, Ile94, Phe104, and Ile117. This site includes most of the hydrophobic pocket of MD-2, facilitating CH−π and van der Waals interactions with the ligand. Mangiferin is the simpler example of this, by introducing the bigger tricyclic scaffold into the MD-2 hydrophobic pocket and orienting the β-C-glycoside moiety toward the more hydrophilic region (Figure 4D). Compound **32** and its more complex derivatives **151** and **168** also bind to this site, primarily through π-stacking interactions with residue Phe76 and an additional interaction with Phe151 (Figure 4E). It is worth noting that the smallest compound **32** mainly interacts with Phe151 and establishes nonpolar contacts with Leu61, Ile63, and Phe76, but lacks the interactions of compounds **151** and **168**, whose aliphatic chains allow them to establish more hydrophobic interactions with deeper residues of this hydrophobic cavity, such as Phe76, Ile94, or Phe104. On the other hand, for the smaller compound **3p**, which has a mainly polar nature, many different binding poses are predicted outside the MD-2 pocket with an unclear clustering of these docked poses. This suggests the coexistence of alternative binding modes, probably by attaching to polar patches of the TLR4/MD-2 interface, whose assessment would require further studies. **Site III** is formed by the same amino acids of site II, and polar amino acids placed above this hydrophobic pocket, on the MD-2 outer rim (Arg96, Asp100, and Tyr102 from MD-2, and Arg264 and Asn339 from TLR4), which contribute to the formation of polar interactions. CA and Mitoxantrone are placed in this region in a tweezer-like shape, introducing one of their side chains into the hydrophobic cavity, while the other is placed toward this polar outer rim, retaining the nonpolar central scaffold in contact with Leu61, Ile63, Phe76, or Ile94 (Figure 4F).

According to our data, the introduction of an aromatic scaffold, small if it is polysubstituted or larger if it is monosubstituted, seems to improve the affinity for site I. Regarding the substitution pattern of this ring, the introduction of a nonpolar chain of 4 to 7 atoms, even with a terminal phenyl-like aromatic moiety, could effectively improve the affinity for site II, whereas the introduction of a 4–7 unit chain with a terminal polar group seems to improve the affinity for site III. Thus, the inclusion of those modifications, alone or in combination, in the design of new molecules could improve their affinity for MD-2 (Figure 5).

**Figure 5:**
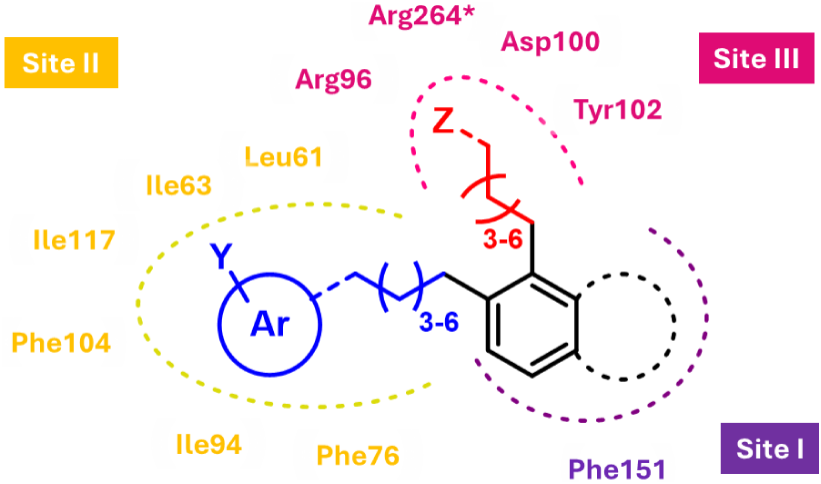
Pharmacophore proposal is based on our docking studies. Y represents small substituents (*e.g.,* CH_3_, CF_3_, halogens), and Z represents only polar substituents.

## Discussion

Out of a multitude of anti-inflammatory NCs studied in the literature, in this critical perspective, we selected a set of molecules with original scaffolds that have been stringently proven to be TLR4 antagonists showing drug-like properties (lack of toxicity, good pharmacodynamic and pharmacokinetic in animal experiments). The NCs here selected are active in high μM concentrations, and in some cases, a dramatic increase in activity (pharmacodynamic) and also in solubility, bioavailability, and pharmacokinetic properties has been observed upon chemical modification of the parent structures. DHA semisynthetic derivatives (compounds **2k** and **5n**) and Dysodensyol K analogues (compounds **151**, **168**, **32**, and **3p**) showed improved activity compared to their natural precursors as TLR4 antagonists, proving that covalent modifications are effective in improving both pharmacodynamic and pharmacokinetic properties.

Docking analysis performed through a homogeneous methodology is essential to compare the binding sites of different classes of molecules and extrapolate a common pharmacophore or fingerprints. Having a homogeneous dataset of docked molecules is fundamental in the drug discovery process to access novel scaffolds and produce new effective drugs. According to our calculations for this small molecule library, the presence of an aromatic ring favors the interaction with Phe151, which contributes to the positioning of the ligand inside the MD-2 pocket. Additionally, an aliphatic side chain with a terminal polar group directs this chain toward the upper polar rim, while the presence of a nonpolar side chain directs that chain into the hydrophobic pocket of MD-2. The presence of these additional side chains provides anchorage points that correlate with improved affinity of the small molecule inserted inside MD-2. These interesting conclusions should be further analyzed with expanded libraries to derive more precise information useful for the design of small-molecule TLR4 antagonistic activity inspired in NC.

Modification of the structures could lead to improved selectivity toward TLR4, decreasing off-target effects and enhancing activity toward the receptor. Despite that further studies are required to have a full understanding of TLR4 antagonism requirements, few considerations could be made from the data presented here. Data suggest that, among the molecules interacting with both MD-2 and TLR4, the ones having the capability to access more lipophilic environments are more active (e.g., DHA and compound **2k**), as they are better accommodated in the lipophilic pocket of MD-2. These hydrophobic interactions are mainly represented by aromatic interactions (π–π stacking/CH−π) with the Phe151 side chain in site I and the Phe76 and Phe104 side chains in site II, and by van der Waals interactions with the side chains of other residues of site II, such as Leu61, Ile63, Ile94, or Ile117. The presence of a hydrophilic moiety is however very important for activity, since it could be interacting with hydrophilic residues from site III, such as MD-2 rim residues Arg96, Arg100, or Tyr102, and with TLR4 residues Arg264 and Asn339, as exemplified in artemisinin–coumarin hybrids and Mangiferin.

Our integrative approach has served as an approximation to identify the preferred binding sites at the target and to extract common chemical features from the presented modulators (i.e., the pharmacophore) by first selecting the most representative compounds from several families of natural products with reported TLR4 antagonist activity and pharmacodynamic/pharmacokinetic properties, second by choosing a proper target protein for the docking calculations, and finally by applying a common and homogeneous docking computational protocol. These efforts to work in an integrated manner can tremendously facilitate the way toward more potent TLR4-targeted antagonists.

## Abbreviation

AD: Alzheimer’s disease
ALI: acute lung injury
ALS: amyotrophic lateral sclerosis
AP1: activator protein 1
ARDS: acute respiratory distress syndrome
CAC: colitis-associated colorectal cancer
CD14: cluster of differentiation 14
CD86: cluster of differentiation 86
CLP: cecal ligation and puncture
CVB3: Coxsackie Virus B3
DAMPs: damage-associated molecular patterns
GSDMD: Gasdermin D
HMGB1: high mobility group box 1
HO-1: heme oxygenase 1
IBDs: inflammatory bowel diseases
IFI16: gamma-interferon-inducible protein 16
IFN: interferon
IL: inteleukin
iNOS: inducible nitric oxide synthase
IRAK4: interleukin-1 receptor-associated kinase 4
IRF3: interferon regulatory factor 3
ITC: isothermal titration calorimetry
JNK: Jun N-terminal kinases
LBP: lipopolysaccharide binding protein
LDL: low-density lipoprotein
LOS: lipooligosaccharide
LPS: lipopolysaccharide
MD-2: myeloid differentiation factor 2
MPO: myeloperoxydase
Myd88: myeloid differentiation primary response 88
NEMO: NF-kappa-B essential modulator
NF-κB: nuclear factor kappa-light-chain-enhancer of activated B cells
NLRP3: NLR family Pyrin 3
NMR: nuclear magnetic resonance
NO: nitric oxide
NOS: nitric oxide synthase
NRCM: neonatal rat cardiomyocytes
PAMPs: pathogen-associated molecular patterns
PBMCs: peripheral blood mononuclear cells
PD: Parkinson disease
PRR: pattern recognition receptor
RA: rheumatoid arthritis
ROS: reactive oxygen species
RSC: rat synovial cells
SAR: structure–activity relationship
SLE: systemic lupus erythematosus
Smad: small mother against decapentaplegic
SPR: surface plasmon resonance
TBK1: TANK-binding kinase 1
TIR: Toll-interleukin receptor
TIRAP: TIR adaptor protein
TLR4: Toll-like receptor 4
TNF: tumor necrosis factor
TRAM: TRIF-related adaptor molecule
TRIF: TIR-domain-containing adapter-inducing interferon-β
Tyr: tyrosine
